# Oxycodone-induced dopaminergic and respiratory effects are modulated by deep brain stimulation

**DOI:** 10.3389/fphar.2023.1199655

**Published:** 2023-06-20

**Authors:** Jason Yuen, Abhinav Goyal, Aaron E. Rusheen, Abbas Z. Kouzani, Michael Berk, Jee Hyun Kim, Susannah J. Tye, Osama A. Abulseoud, Tyler S. Oesterle, Charles D. Blaha, Kevin E. Bennet, Kendall H. Lee, Yoonbae Oh, Hojin Shin

**Affiliations:** ^1^ Department of Neurologic Surgery, Mayo Clinic, Rochester, MN, United States; ^2^ IMPACT—The Institute for Mental and Physical Health and Clinical Translation, School of Medicine, Barwon Health, Deakin University, Geelong, VIC, Australia; ^3^ Medical Scientist Training Program, Mayo Clinic, Rochester, MN, United States; ^4^ School of Engineering, Deakin University, Geelong, VIC, Australia; ^5^ Queensland Brain Institute, The University of Queensland, St Lucia, QLD, Australia; ^6^ Department of Psychiatry and Psychology, Mayo Clinic, Rochester, MN, United States; ^7^ Department of Psychiatry and Behavioral Science, Emory University, Atlanta, GA, United States; ^8^ Department of Psychiatry, University of Minnesota, Minneapolis, MN, United States; ^9^ Department of Psychiatry, Mayo Clinic, Phoenix, AZ, United States; ^10^ Department of Psychiatry, Mayo Clinic, Rochester, MN, United States; ^11^ Division of Engineering, Mayo Clinic, Rochester, MN, United States; ^12^ Department of Biomedical Engineering, Mayo Clinic, Rochester, MN, United States

**Keywords:** substance use disorder, deep brain stimulation, nucleus accumbens, ventral tegmental area, oxycodone, dopamine, rodent

## Abstract

**Introduction:** Opioids are the leading cause of overdose death in the United States, accounting for almost 70,000 deaths in 2020. Deep brain stimulation (DBS) is a promising new treatment for substance use disorders. Here, we hypothesized that VTA DBS would modulate both the dopaminergic and respiratory effect of oxycodone.

**Methods:** Multiple-cyclic square wave voltammetry (M-CSWV) was used to investigate how deep brain stimulation (130 Hz, 0.2 ms, and 0.2 mA) of the rodent ventral segmental area (VTA), which contains abundant dopaminergic neurons, modulates the acute effects of oxycodone administration (2.5 mg/kg, i.v.) on nucleus accumbens core (NAcc) tonic extracellular dopamine levels and respiratory rate in urethane-anesthetized rats (1.5 g/kg, i.p.).

**Results:** I.V. administration of oxycodone resulted in an increase in NAcc tonic dopamine levels (296.9 ± 37.0 nM) compared to baseline (150.7 ± 15.5 nM) and saline administration (152.0 ± 16.1 nM) (296.9 ± 37.0 vs. 150.7 ± 15.5 vs. 152.0 ± 16.1, respectively, *p* = 0.022, *n* = 5). This robust oxycodone-induced increase in NAcc dopamine concentration was associated with a sharp reduction in respiratory rate (111.7 ± 2.6 min^−1^ vs. 67.9 ± 8.3 min^−1^; pre- vs. post-oxycodone; *p* < 0.001). Continuous DBS targeted at the VTA (*n* = 5) reduced baseline dopamine levels, attenuated the oxycodone-induced increase in dopamine levels to (+39.0% vs. +95%), and respiratory depression (121.5 ± 6.7 min^−1^ vs. 105.2 ± 4.1 min^−1^; pre- vs. post-oxycodone; *p* = 0.072).

**Discussion:** Here we demonstrated VTA DBS alleviates oxycodone-induced increases in NAcc dopamine levels and reverses respiratory suppression. These results support the possibility of using neuromodulation technology for treatment of drug addiction.

## Introduction

Opioids are the leading cause of overdose death in the United States, accounting for almost 70,000 deaths in 2020 (Centers for Disease Control and Prevention, 2022). The main side-effect of opioid use is dose tolerance, to which chronic users are particularly susceptible (Ballantyne and Mao, 2003). Current opioid use disorder treatments typically include replacement therapy (e.g., methadone hydrochloride and buprenorphine) and psychosocial interventions (Polydorou et al., 2017; Volkow et al., 2019). Although there has been some success with newer treatments such as buprenorphine-naloxone therapy (Weiss et al., 2011), the relapse rate remains unacceptably high. Indeed, abstinence from opioid use remains less than 30% after 10–30 years (Hser et al., 2015). Deep brain stimulation (DBS) has recently been utilized for the treatment of neuropsychiatric diseases, including depression (Mayberg et al., 2005; Yuen et al., 2021a), Tourette’s syndrome (Baldermann et al., 2016), obsessive-compulsive disorder (Nuttin et al., 1999; Denys et al., 2010; McLaughlin et al., 2021), and drug addiction (Chen et al., 2019; Yuen et al., 2022a). With promising findings from recently published studies, there are ongoing clinical trials to assess the long-term effect of DBS for treating drug addiction, particularly focusing on the nucleus accumbens (NAc) as a target (Zhou et al., 2011; Valencia-Alfonso et al., 2012; Chen et al., 2019; Clinical Trials, 2019; Qu et al., 2019; Zhang et al., 2020; Mahoney et al., 2021).

However, electrical stimulation is inherently non-selective in what neuronal elements and terminals are activated. Thus, Nac DBS is releasing many different neurotransmitters in the Nac (e.g., glutamate, dopamine, acetylcholine) which does not address what neurotransmitter may be the most relevant in mitigating addiction. In opioid use disorder, dopamine is one of the key monoamine neurotransmitters that mediates incentive-motivation which plays an important role in drug reinforcement and compulsive drug-seeking behaviors. Opioids are known to stimulate µ-opioid receptors on GABAergic nerve terminals synapsing on dopamine neuronal cells in the ventral tegmental area (VTA) producing both dopamine-dependent and dopamine-independent positive reinforcement (Ting and van der Kooy, 2012). Activation of these presynaptic µ-opioid receptors reduces GABAergic inhibition of dopamine neurons, leading to an increase in dopamine cell firing and production and release of dopamine in the NAc.

Given that opioid driven dopamine release in the NAc is considered an important factor in opioid use disorder, rather than stimulating the NAc the present study instead examined the effects of VTA DBS, which would specifically interfere with opioid-induced disinhibition of dopamine cell firing by GABA interneuronal cells in the VTA as a means of attenuating the effects of opioids. Normally, the prefrontal cortex provides negative feedback to overcome drives to perform unsafe or unwise activities. However, this feedback system becomes compromised with repeated drug administration and addiction (Kosten and George, 2002; Barrot, 2015). Repeated administration results in drug tolerance, characterized by reduced dopamine release after normally rewarding activities. This leads to dependence and compulsive drug-seeking behavior (Kosten and George, 2002). The neurocircuitry involved in the behavioral changes may be better understood by measuring dopamine accurately, particularly during therapeutic treatments. In addition, dopamine has parallel effects on respiration, generally enhancing CO_2_ and pH dependent respiratory drive (Lalley, 2008). To our knowledge, no studies have investigated the efficacy of DBS in attenuating opioid-induced respiratory depression.

Microdialysis is the current gold standard to measure dopamine release into the NAc extracellular space (Saigusa et al., 2021). However, newer electrochemical techniques such as multiple cyclic square wave voltammetry (M-CSWV) can measure tonic (basal) dopamine levels with higher spatio-temporal resolution and causes less trauma to the brain tissues of interest (Oh et al., 2018; Rusheen et al., 2020; Yuen et al., 2021b).

In the current proof-of-concept study, we hypothesized that VTA DBS would provide modulation of the oxycodone effect on tonic dopamine levels, as well as its ability to reduce the suppressive effect on respiration. To achieve this, we first elucidated oxycodone-induced dopaminergic changes *in vivo* using M-CSWV and simultaneously measured the respiratory rate. Then, we performed DBS of the VTA while simultaneously measuring oxycodone-induced changes in dopamine extracellular levels in the core of the nucleus accumbens (NAcc) and respiratory rate changes caused by acute intravenous oxycodone administration.

## Material and methods

### Animal subjects

Adult male Sprague-Dawley rats (*n* = 10, weighing 250–300 g; Envigo, IN, United States) were used for this study. Rats were kept in group housing in an Association for Assessment and Accreditation of Laboratory Animal Care International (AAALAC) accredited vivarium following a standard 12-h light/dark cycle at constant temperature (21°C) and humidity (45%) with *ad libitum* food and water. Animal studies were approved by the Institutional Animal Care and Use Committee (IACUC), Mayo Clinic, Rochester. The NIH Guide for the Care and Use of Laboratory Animals guidelines (Department of Health and Human Services, NIH publication No. 86-23, revised 1985) were followed for all aspects of animal care.

### Dopamine sensing microelectrode fabrication

CFMs were fabricated using an established standardized CFM design at Mayo Clinic (Chang et al., 2013; Oh et al., 2016). Further details, including preparation of the Ag/AgCl counter-reference electrode can be found in our group’s previous publications (Yuen et al., 2021b; Yuen et al., 2022b). CFMs were pretested in TRIS buffer prior to coating deposition with a PEDOT:Nafion deposition solution (Vreeland et al., 2015), which minimized the effect of biofouling *in vivo*.

### Implantation of recording and stimulating electrodes

Each rat was anesthetized with urethane (1.5 g/kg i.p.; Sigma-Aldrich, St Louis, MO, United States) and administered buprenorphine (0.05–0.1 mg/kg s.c., Par Pharmaceutical, Chestnut Ridge, NY, United States) for analgesia. Following anesthesia, the rat was placed in a stereotaxic frame (David Kopf Instruments, Tujunga, CA, United States). Respiratory rate (RespiRAT, Intuitive Measurement Systems, AZ, United States) and hind-paw and tail pinch were used to monitor the physiological state and depth of anesthesia, respectively. RespiRAT is a pressure monitor that only counts the peaks of pressure to mitigate interference from noise, and automatically adjusts the detection threshold with the amplitude of the incoming data. Using a standard rat brain atlas (Paxinos and Watson, 2007), three trephine holes were drilled, the first for placement of a CFM into the NAcc (all coordinates from bregma: AP 1.2 mm, ML 2.0 mm, DV 6.5–7.5 mm from dura), the second for a stimulating electrode into the VTA (twisted bipolar stimulating electrode—Plastics One, MS 303/2, Roanoke, VA, United States, with the tips separated by ∼1 mm; AP −5.3 mm, ML 0.9 mm, DV 7.5–9 mm from dura), and a third for an Ag/AgCl reference electrode into the contralateral cortex (Clark et al., 2010).

### Recordings and stimulation parameters

The depths of the stimulating electrode in the VTA and CFM in the NAcc were first adjusted to obtain a robust stimulation-evoked dopamine signal as measured by fast scan cyclic voltammetry (FSCV) (−0.4 to 1.3 V sweep; 10 Hz). Stimulation parameters were biphasic pulses at 60 Hz, 2 ms pulse width, 0.2 mA, and 2 s duration. Stimulation and FSCV were both performed using the WINCS Harmoni system (Lee et al., 2017), a wireless stimulation and neurochemical sensing system.

Once the optimal electrode depths were identified, the system was switched to the M-CSWV sensing technique (Oh et al., 2018). After at least 60 min of stabilization, the rats were treated according to one of the two experiments. For Experiment 1 (N = 5), i.v. saline (1 mL/kg) was first administered as a negative control. After 30 min, i.v. oxycodone (provided by National Institute on Drug Abuse, Bethesda, MD; 2.5 mg/kg dissolved in 0.5 mL of normal saline) was administered (infused over 2 min via a cannula inserted into the tail vein) and the rat was further observed for another 30 min.

Experiment 2 consisted of the stimulation group (separate set of animals; N = 5), VTA biphasic pulse stimulation was applied at 130 Hz (0.2 ms pulse width, 0.2 mA) continuously for 60 min. The delivered stimulation was interleaved with the M-CSWV recording to minimize artifacts. Once the signal was restabilized to a new plateau (after 30 min of stimulation), i.v. oxycodone was administered (dose and method as above) while stimulation and recording continued. After another 30 min of observation, the stimulation was turned off. The animal was observed for another 30 min post-stimulation before being euthanized using Fatalplus injection (pentobarbital 390 mg/mL i.p.; 10 mL).

Saline administration during VTA stimulation was shown to have no effect on tonic dopamine levels in our previous study and was therefore not repeated here [Figures 4B, 5B of (Yuen et al., 2023)].

### Calibration of electrodes

After experimentation, changes in dopamine release recorded from individual CFMs were calibrated *in vitro* with dopamine solutions of different known concentrations. This was performed in a similar manner to previously described procedures (Oh et al., 2018).

### Statistical analysis

For comparison between different dopamine concentrations, repeated-measures one-way (Experiment 1) and two-way (Experiment 2) ANOVA tests were performed. The levels for comparison were all calculated by averaging over 10 data points, i.e., 100 s. In Experiment 1, the levels include: “Baseline” (centered at 5 min prior to saline injection), “Saline” (same post-administration time interval as the “Oxycodone” measurement for fair comparison), and “Oxycodone (Peak)” (centered at the peak of dopamine levels after oxycodone was administered). Post-hoc two-tailed paired *t*-tests were also conducted in the Experiment 1 between sequential measurements (Baseline vs. Saline; Saline vs. Oxycodone).

In Experiment 2, two-way ANOVA test was performed across: “Baseline” (centered at 5 min prior to start of stimulation), “Stimulation” (centered at 5 min prior to administering oxycodone); “Oxycodone” (centered at the peak of dopamine levels after oxycodone was administered), and “post-stimulation” (centered at 15 min after stimulation stops).

For comparison between respiratory rates, two-tailed paired *t*-tests were also conducted. Respiratory rates are measured by calculating the moving average over 30 s. Measurements were taken approximately 1/54 s apart. The levels for comparison were calculated by averaging over 10 data points. In Experiment 1, a comparison was made between the levels post-saline (same post-administration time interval as the post-oxycodone measurement for fair comparison) and the levels post-oxycodone (centered at troughs). Likewise, in Experiment 2, a comparison was made between the levels post-stimulation (10 min prior to oxycodone administration) and the levels post-oxycodone (centered at troughs).

Statistical analyses were performed and graphs were constructed using PRISM 8 (GraphPad). All error bars and shaded areas are represented as S.E.M. Statistical significance was set at *p* < 0.05. No Bonferroni corrections to *t*-tests were applied as sequential comparisons were set up for testing a pre-planned hypothesis (sequential changes).

## Results

**FIGURE 1:**
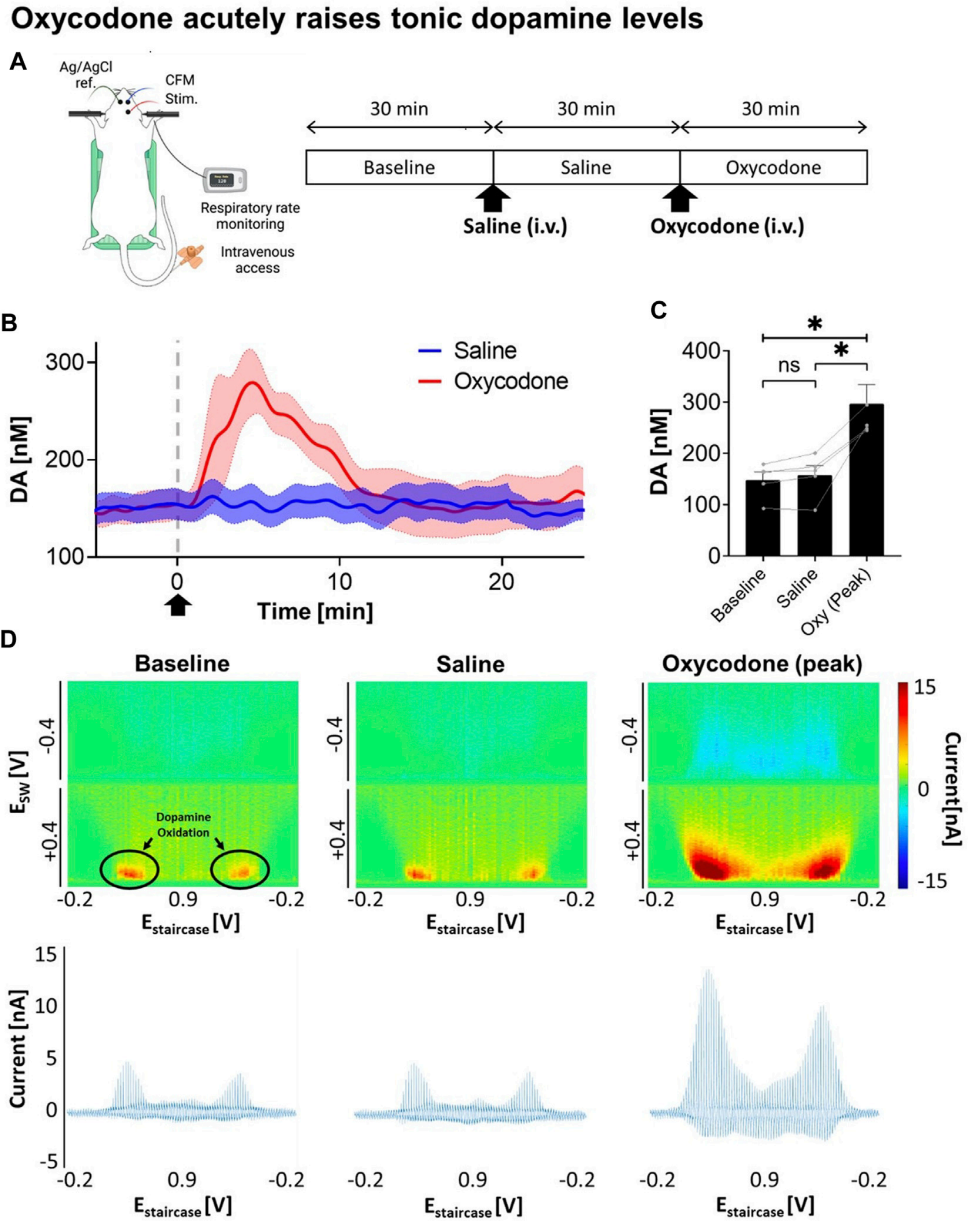
Changes in NAcc tonic dopamine concentrations after saline and oxycodone. **(A)** Experimental design and procedure. All electrodes were implanted stereotactically into the brain of urethane-anesthetized rats and positioned at the optimal depth. Tonic dopamine levels in the NAcc were recorded throughout the experiments. Saline and oxycodone were administered i.v. accordingly. **(B)** Rapid increase in dopamine was seen after i.v. oxycodone administration (2.5 mg/kg) compared to i.v. saline (1 mL/kg). Arrow denotes time of oxycodone administration. **(C)** One-way ANOVA demonstrated significant changes among the levels (*F* = 12.80; *p* = 0.0224). *Post-hoc* analysis showed saline administration did not significantly alter tonic dopamine levels (+1.2 ± 4.4 nM, +0.8%, N = 5 rats, *p* = 0.1047); whereas oxycodone rapidly increased dopamine levels within ∼5 min of administration (+145.0 ± 41.9 nM, +95%, N = 5 rats, *p* = 0.0251). * denotes *p* < 0.05. **(D)** Representative high dimensional color plots and matched voltammogram at baseline, and after saline and oxycodone administration, respectively. The circle denotes dopamine oxidation on the color plot measured by M-CSWV. Figure partly created with BioRender.com.

To determine the viability of VTA DBS as a potential therapy for opioid intoxication, dopamine levels were first monitored in response to oxycodone injection. A saline injection was performed before the oxycodone injection as shown in Figure 1A. Using M-CSWV, it was demonstrated that there was a significant increase in NAcc tonic dopamine levels after oxycodone administration (Figure 1B). A one-way ANOVA demonstrated significant changes among the baseline, saline, and oxycodone (peak) dopamine levels (*p* = 0.0224). Post-hoc analysis showed saline did not significantly alter tonic dopamine levels (+1.2 ± 4.4 nM, +0.8%, N = 5 rats, *p* = 0.1047) (Figure 1C). In contrast, oxycodone rapidly increased tonic dopamine levels within 10 min from 157.7 ± 41.4 nM to 296.9 ± 82.8 nM (*n* = 5 rats, *p* = 0.0251). This is also highlighted in the high dimensional color plots, and their voltammograms in Figure 1D Higher dopamine concentrations are displayed as more intense red.

**FIGURE 2:**
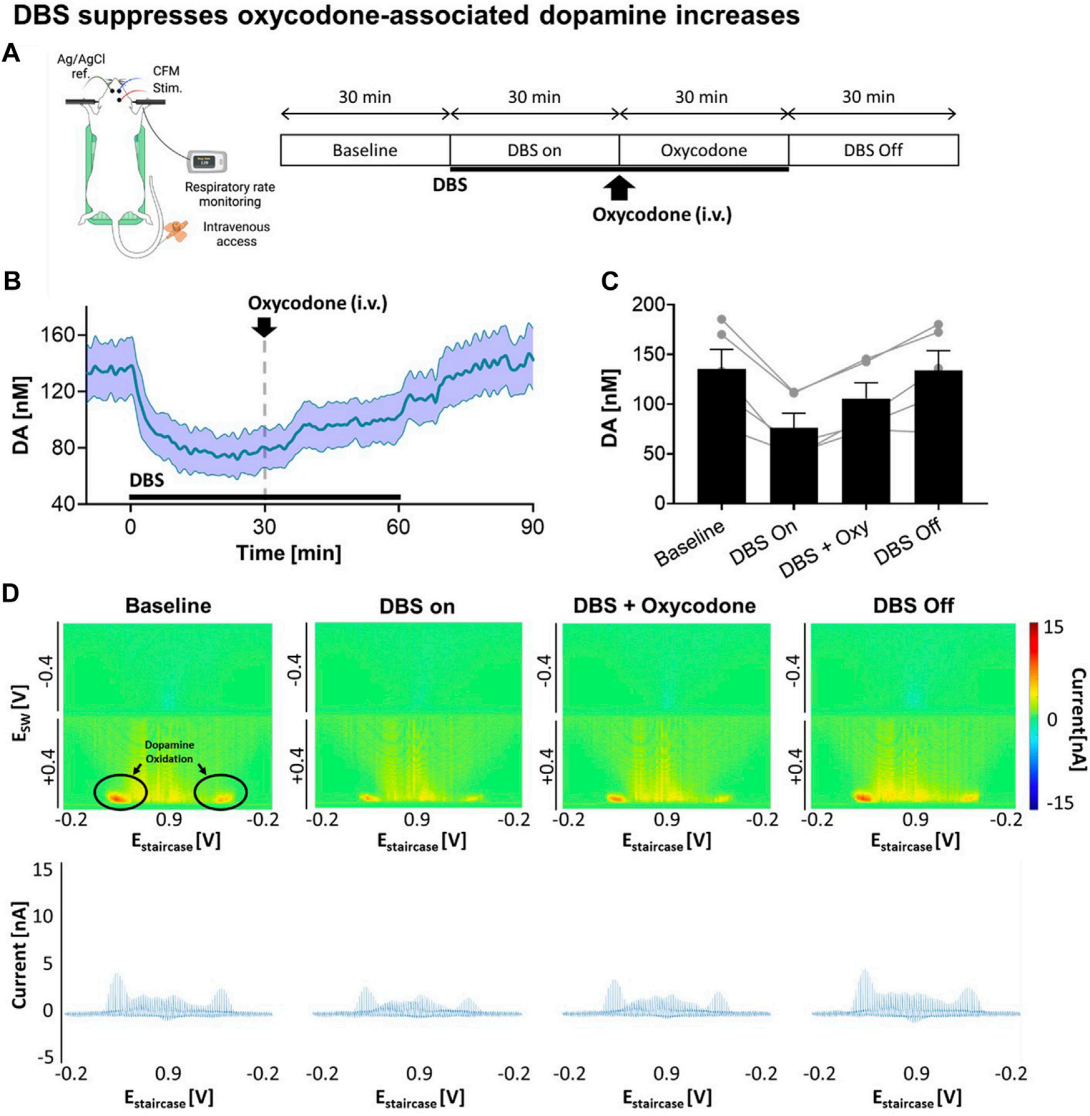
Stimulation suppresses oxycodone-associated dopamine increases. **(A)** Experimental design and procedure. All electrodes were implanted stereotactically into the brain of urethane-anesthetized rats and positioned at the optimal depth. Tonic dopamine levels in the NAcc were recorded throughout the experiments. VTA DBS (biphasic 130 Hz, 200 µs pulse width, 0.2 mA) was applied (black bar). Oxycodone (2.5 mg/kg) was given intravenously. **(B)** Stimulation suppressed tonic dopamine levels. The levels increased after oxycodone administration but remained below the pre-stimulation baseline. After cessation of stimulation, the levels returned to approximately baseline. Black bars represent the stimulation period. The red arrow denotes drug administration. **(C)** Two-way ANOVA test demonstrated stimulation [*F*(1,4) = 30.48; *p* = 0.0053] and oxycodone [*F*(1,4) = 21.81; *p* = 0.0095] both had a statistically significant effect on tonic dopamine levels (N = 5 rats). **(D)** Representative high dimensional color plots and matched voltammogram corresponding to the time points. The circle and arrow denote the site of dopamine oxidation on the color plot measured by M-CSWV. Figure partly created with BioRender.com.

Next, we explored how VTA DBS affects tonic dopamine levels in the NAcc, including after oxycodone administration. As shown in Figure 2A, continuous VTA DBS was applied for 1 h. During the stimulation oxycodone (i.v.) was administered while monitoring changes in tonic dopamine levels. After 30 min, DBS was turned off and tonic dopamine levels were recorded for an additional 30 min. With VTA DBS, tonic dopamine levels in the NAcc decreased by 43.8%, from 135.4 ± 43.8 nM to 76.1 ± 32.7 nM (Figure 2B). These levels were augmented by oxycodone administration to 105.8 ± 35.0 nM (+39.0%) but the levels did not return to baseline until stimulation was switched off. After VTA DBS was turned off, tonic dopamine levels gradually returned to baseline. ANOVA analysis demonstrated stimulation [*F*(1,4) = 30.48; *p* = 0.0053] and oxycodone [*F*(1,4) = 21.81; *p* = 0.0095] both had a statistically significant effect on tonic dopamine levels (Figure 2C). Interaction between the two factors was also found to be significant [*F*(1,4) = 130.5; *p* = 0.0003]. This was also reflected in the less intense red color on the color plots (Figure 2D).

**FIGURE 3:**
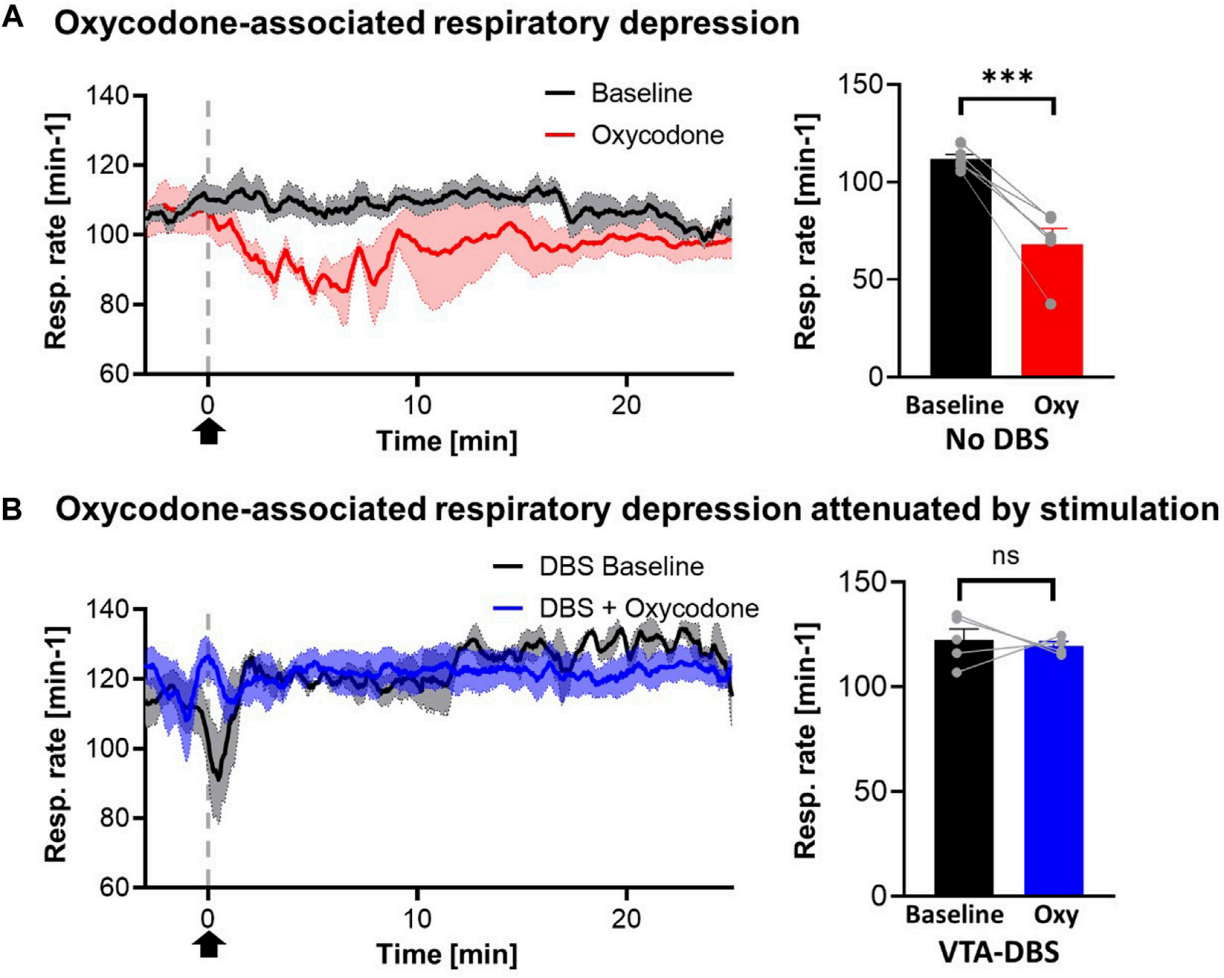
Oxycodone-associated respiratory depression attenuated by stimulation. **(A)** Respiratory rates reduced acutely after i.v. oxycodone (2.5 mg/kg) (red line) but not with i.v. saline (1 mL/kg) (blue line). However, with stimulation, giving oxycodone did not cause a significant drop in respiratory rates (black line). Arrow denotes time point where drug was administered. **(B)** Statistical analysis of respiratory rates demonstrated significant changes in cases where no stimulation was applied (*p* < 0.001) but this was reversed when stimulation was applied (*p* = 0.072) (N = 5 for each group). *** denotes *p* < 0.001.

One consideration was that VTA DBS may alleviate the respiration-suppressing effects of acute opioid administration. Respiration rate was monitored and compared for saline and oxycodone treatment in the absence of VTA DBS, and oxycodone treatment during VTA DBS (Figure 3A). Oxycodone was shown to decrease the respiratory rate by 36.6% relative to baseline (*p* < 0.001). VTA stimulation partially alleviated respiratory suppression from 36.6% to 13.4% relative to baseline (*p* = 0.072) (Figure 3B). The clear trough in respiratory rate secondary to oxycodone was also less visible when stimulation was applied. Although there appears to be an increase in respiratory rates after VTA DBS was initiated (prior to oxycodone), compared to baseline just before VTA DBS, there was no statistical differences (*p* = 0.148).

## Discussion

The present study measured tonic dopamine levels in the NAcc and accompanying respiratory rate following i.v. oxycodone administration. The facilitatory increase in dopamine levels is consistent with previous microdialysis studies with opioids (Vander Weele et al., 2014; Saigusa et al., 2017). The rise in dopamine levels is also similar to our previous studies on the effect of i.v. cocaine on accumbal tonic dopamine levels, and is concordant with a critical role of dopamine across addictive agents (Solinas et al., 2019; Yuen et al., 2021b). In addition, VTA DBS was also shown to significantly suppress baseline tonic dopamine levels in the NAcc and can further significantly attenuate the ability of oxycodone to elevate dopamine levels, which is consistent with our previous study with cocaine (Yuen et al., 2023). The respiratory rate depression following oxycodone administration was also partially reversed by VTA DBS.

While FSCV has been utilized in studying short-term effects of opioids on *phasic* (burst) dopamine release (Vander Weele et al., 2014), to our knowledge, no studies have used electrochemical techniques to study the effects of opioids on *tonic* dopamine concentrations.

### Potential mechanism of action of VTA DBS

Continuous application of VTA DBS resulted in a consistent suppression of NAcc tonic dopamine levels. This also attenuated the facilitatory effects of oxycodone on tonic dopamine levels, with dopamine levels returning to baseline after cessation of DBS. The depletion of presynaptic dopamine vesicular stores may cause this reversible effect. In this case, an initial peak would be expected. Although this was not apparent in the averaged data (Figure 2B), an initial and brief peak increase was indeed seen in three out of five rats (60%) that underwent stimulation. We defined an initial peak to be one that occurs within 1 min (6 MCSWV readings) that is at least more than 2 standard deviations of the 10 averaged points used to calculate the baseline prior to stimulation. The average increase in dopamine among the three rats was 14.4 ± 3.7 mM. This is consistent with the phasic (evoked) increases of dopamine in the NAcc (measured by FSCV) that were seen with brief (2 s) stimulation of the VTA during the targeting progress. This may not be seen universally as the M-CSWV technique has a time resolution of 10 s and if the release of dopamine occurred prior to the following measurement, the peak may be much smaller. Previous studies have shown that prolonged intermittent VTA stimulation was associated with reduced dopamine levels in the NAc, likely due to depletion of accumbal presynaptic dopamine vesicular stores or autoreceptor activation (Hernandez et al., 2006; Hernandez and Shizgal, 2009). This is consistent with the phenomenon seen in the current study. Another explanation may be activation or inhibition of other inhibitory and excitatory neurotransmitters. Examples include GABA (inhibitory), dopamine (inhibitory), glutamate (excitatory), and acetylcholine (facilitatory), all of which have been found in the VTA in microdialysis studies (Westerink et al., 1996; Lester et al., 2010). It is well known that opiates increase firing activity of dopamine neurons at least in part by decreasing the inhibitory effect of GABA via activation of µ-opiate receptors on GABAergic interneuron terminals in the VTA, and glutamate transmission is required for morphine’s effect on dopamine cells (Barrot, 2015; Saigusa et al., 2021). Therefore, DBS may exert its effect on extracellular dopamine levels via modulation of these other neurotransmitters.

One further possibility is that continuous electrical stimulation of the VTA may induce a relatively rapid depolarization block (DB) similar to chronic antipsychotic treatments that, in turn, decrease tonic DA levels in the NAcc (Lane and Blaha, 1987; Grace et al., 1997; Boye and Rompre, 2000; Hernandez et al., 2016). These hypotheses can be investigated by further studies using other pharmacological tests and measuring techniques such as amperometry.

### Potential clinical applications

VTA DBS is currently being used as an experimental treatment for treatment-refractory cluster headache (Akram et al., 2016). Interestingly, it has been shown that elevation of peripheral dopamine levels is implicated in cluster headache pathophysiology, suggesting cluster headaches may be a consequence of overactivity of the dopaminergic and autonomic systems (D'Andrea et al., 2006; D'Andrea et al., 2019). Therefore, the therapeutic effect of VTA DBS on cluster headache may be explained by its suppressive effect on forebrain dopamine levels.

With regards to the potential applicability of DBS for substance use disorder, no human studies have been reported using VTA DBS. In preclinical studies, one study demonstrated continuous optogenetic stimulation of VTA dopaminergic neurons can reduce ethanol self-administration in mice (Bass et al., 2013). Although optogenetic and electrical stimulation have different underlying mechanisms of action, one study demonstrated that both modalities could activate similar brain regions (Weidner et al., 2020).

In the current study, the NAc *core* was chosen as the main target for dopamine measurements for two main reasons. First, from microdialysis studies, extracellular dopamine in the core was higher than that in the shell, whereas norepinephrine levels (a potential electroactive interferent) were higher in the shell (McKittrick and Abercrombie, 2007). Second, these are acute experiments where the animals are exposed to the drug for the first time. It has been suggested that the NAcc underlies the phenomenon of initial acquisition of drug taking, cue-elicited drug seeking behavior, and impulsive choices (Di Chiara, 2002; Salgado and Kaplitt, 2015). Although we did not perform comparison studies using the accumbens shell subregion, it remains an interesting target for future studies using opioids in combination with VTA DBS. VTA DBS may modulate drug-seeking behavior by suppressing dopamine levels in the NAcc. This may also be applicable to hyperdopaminergic pathologies such as mania, schizophrenia, and dopamine dysregulation syndrome (Berk et al., 2007; Grace, 2016; Ashok et al., 2017).

Consistent with previous microdialysis studies (Westerink et al., 1996; Lester et al., 2010), oxycodone induced significant increases in NAcc dopamine levels, which likely account for the initiating steps in the pathophysiology of opioid use disorder. The suppression of NAcc dopamine levels appeared reversible within 60 min of DBS but this may not be the case for longer stimulation duration or repeated stimulations. Given dopamine is associated with both drug-related and pharmacological rewards, depression of dopamine levels in a normal dopaminergic state may theoretically lead to anhedonia and depression. Therefore, more studies need to be conducted before widely implementing this potential therapeutic option. In addition, the present study suggests that intermittent or continuous measurement of tonic dopamine levels may serve as a potential biomarker for closed-loop DBS for substance use disorder. However, the risks of implanting a separate sensing electrode and the ethics of such interventions should be considered (Lo et al., 2023).

The cases of opioid overdose in the United States have more than quadrupled between 2000 and 2020 and, without further intervention, is expected to double by 2029 (Humphreys et al., 2022). Therefore, it is of interest that the current study demonstrated that VTA DBS may be used to reduce the effects of oxycodone and therefore potentially prevent overdose deaths. Given the volume of tissue affected by electrical stimulation, it is highly likely that other brain structures, such as descending white matter fibers that control the motor aspect of respiratory effort, were co-stimulated, leading to the observed attenuating effect on oxycodone-associated respiratory suppression. Several studies using intracranial self-stimulation also showed that DBS can alter the physiological parameters such as the respiratory rate, demonstrating the complex interplay between brain stimulation and the autonomic nervous system (Malmo, 1963; Angyan, 1981; Burgess et al., 1993). One more possible explanation is that stimulation of the VTA would likely also stimulate the substantia nigra (SN) given its proximity to the VTA. There are known anatomical connections between the SN compacta and respiratory control centers, including a direct SNc pathway to the pre-Bötzinger complex (Zhou et al., 2011). The pre-Bötzinger complex interneurons have direct connections to caudal areas containing bulbospinal neurons that are directly involved in generating rhythm to the premotor neurons that transmit oscillatory drive to spinal respiratory motoneurons (Mahoney et al., 2021). Indeed, endogenous activation of D1Rs acts against opioid depression of the respiratory network (Qu et al., 2019). Nevertheless, to our knowledge, this is the first study that demonstrated DBS can attenuate the respiratory effect of opioids. Further optogenetic studies that focus on particular neurons such as dopamine or glutamate neurons, may be used to further discern the target that led to this effect. Also, tractography, particularly in larger animal studies, may be used to map the relationship between the volume of tissue activated by DBS and white matter tracts near the VTA.

Limited historical precedence or evidence implicates DBS as a potential procedure for respiration modulation. One study showed subthalamic nucleus stimulation may improve upper respiratory and laryngeal control in Parkinson’s patients (Hammer et al., 2010), but no lower respiratory effect (intercostal muscles and diaphragm) was measured. Our results suggest that, in addition to opioid overdose, diseases characterized by respiratory insufficiency or failure, such as intractable sleep apnea, may be treated by DBS.

### Experimental limitations and future studies

This proof-of-concept preclinical study utilized anesthetized, naïve rats as subjects rather than a chronic addiction model. Although a relatively high acute dose of oxycodone was administered that induced a clinically corollary of respiratory depression, there were no measures for addictive behaviors such as drug-seeking and compulsion. Future studies in awake animals, particularly in drug self-administration paradigms, will be required to validate the dopaminergic effects, as well as behavioral and respiratory benefits.

While dopamine plays a key role in the process of drug addiction, there are also studies which showed opioids may have a dopamine-independent reward mechanism (Ettenberg et al., 1982; Pettit et al., 1984; Nader and van der Kooy, 1997; Hnasko et al., 2005). Therefore, how much VTA DBS may alter the behavioral changes should be further studied.

## Conclusion

The present study demonstrated that M-CSWV served as a reliable tool to measure changes in tonic extracellular dopamine levels in the NAcc following acute i.v. oxycodone administration. The observation that VTA DBS at least partially reversed the opioid-induced increase in NAcc dopamine levels and respiratory rate depression provides the basis for exploring the use of DBS to treat opioid use disorder and overdose.

## Data Availability

The raw data supporting the conclusion of this article will be made available by the authors, without undue reservation.

## Abbreviations

AAALAC, Association for Assessment and Accreditation of Laboratory Animal Care International; CFM, carbon fiber microelectrod; DBS, deep brain stimulation; FSCV, fast scan cyclic voltammetry; IACUC, Institutional Animal Care and Use Committee; M-CSWV, multiple cyclic square wave voltammetry; NAc, nucleus accumbens; NAcc, nucleus accumbens core; VTA, ventral tegmental area.
